# Anti-EGFR Targeted Multifunctional I-131 Radio-Nanotherapeutic for Treating Osteosarcoma: In Vitro 3D Tumor Spheroid Model

**DOI:** 10.3390/nano12193517

**Published:** 2022-10-08

**Authors:** Suphalak Khamruang Marshall, Boonyisa Saelim, Maneerat Taweesap, Verachai Pachana, Yada Panrak, Naritsara Makchuchit, Passara Jaroenpakdee

**Affiliations:** 1 Department of Radiology, Faculty of Medicine, Prince of Songkla University, Songkhla 90110, Thailand; 2 Molecular Imaging and Cyclotron Center, Division of Nuclear Medicine, Department of Radiology, Faculty of Medicine, Prince of Songkla University, Songkhla 90110, Thailand

**Keywords:** 3D spheroid, cancer treatment, doxorubicin, EGFR targeted therapy, epidermal growth factor receptor, I-131, multiple therapeutic, osteosarcoma, radiolabeled nanoparticles, targeted drug delivery

## Abstract

The systemic delivery of doxorubicin (DOX) to treat osteosarcoma requires an adequate drug concentration to be effective, but in doing so, it raises the risk of increasing organ off-target toxicity and developing drug resistance. Herein, this study reveals a multiple therapeutic nanocarrier delivery platform that overcomes off-target toxicity by providing good specificity and imparting enhanced tumor penetration in a three-dimensional (3D) human MG-63 spheroid model. By synthesizing PEG-PLGA nanoparticles by the double emulsion method, encapsulating DOX and Na^131^I in the inner core, and conjugating with an epidermal growth factor receptor (EGFR) antibody, it is intended to specifically target human MG-63 cells. The nanocarrier is biocompatible with blood and has good stability characteristics. Na^131^I encapsulation efficiency was >96%, and radiochemical purity was >96% over 96 h. A DOX encapsulation efficacy of ~80% was achieved, with a drug loading efficiency of ~3%, and a sustained DOX release over 5 days. The nanocarrier EGFR antibody achieved a ~80-fold greater targeting efficacy to MG-63 cells (EGFR+) than fibroblast cells (EGFR−). The targeted multiple therapeutic DIE-NPs have a higher penetration and uptake of Na^131^I to the 3D model and a ~3-fold higher cytotoxicity than the DOX monotherapy (D-NPs). The co-administration of DOX and Na^131^I (DIE-NPs) disrupts DNA repair and generates free radicals resulting in DNA damage, triggering the activation of apoptosis pathways. This leads to inhibition of MG-63 cell proliferation and promotes cell cycle arrest in the G0/G1 phase. Furthermore, the PEGylated anti-EGFR functionalized DIE-NPs were found to be biocompatible with red blood cells and to have no adverse effects. This anti-EGFR targeted multifunctional I-131 radio-nanotherapeutic signifies a customizable specific targeted treatment for osteosarcoma.

## 1. Introduction

Osteosarcoma is the most common primary bone tumor in developing teenagers and most often affects weight-bearing long bones such as the distal femur and proximal tibia. It is an uncommon malignant bone tumor differentiated by malignant cells forming an unmineralized osteoid matrix. The global incidence of deaths per year is estimated at 4.4 per million people, and the 5-year survival rate for patients with localized osteosarcoma non-invasive stage is 64.5 ± 8.1%. However, in the advanced stage with distant metastases, the survival rate is 16.2 ± 7.9% [1]. Those with localized osteosarcoma cancers have a 5-year relative survival rate of 70%, whereas those with distant metastases have a survival rate of under 30% [2]. Despite breakthroughs, the clinical manifestations of osteosarcoma are unclear, and the tumor can grow and spread rapidly, impairing the patient’s quality of life. Although the success rate of surgical resection against localized osteosarcoma is reported as less than 20%, when surgery is combined with chemotherapy, the success rate rises considerably to approximately 70% [3]. Another critical point is that metastasis still remains a significant challenge. In particular, lung metastases from osteosarcoma are the second primary cause of cancer death in adolescents [4]. 

Doxorubicin (DOX) is used extensively in treating osteosarcoma [5,6]. However, attaining an adequate concentration in tumor tissue after systemic delivery is difficult. Furthermore, osteosarcoma therapy typically lasts for 6–8 months, and systemic dosing raises the risk of toxicity to the bone marrow, mucosal tissue, alopecia, cardiotoxicity, and congestive heart failure [7]. In addition, DOX resistance in osteosarcoma has been found in a number of investigations, responding only to high dosages and rapidly developing resistance, as shown by the poor 5-year survival rate of <20% [8]. Moreover, the treatment of osteosarcoma is also physically hindered due to the existence of denser osteoid, resulting in the common side effects of chemotherapeutics systemic administration [9]. Furthermore, the dense extracellular matrix of solid osteosarcoma tumors restricts interstitial diffusion of systemically injected chemotherapeutics. Elevated interstitial fluid pressure caused by the densely packed osteosarcoma tumor cells reduces the uptake efficacy of therapeutic agents [10], and as a consequence, the reduced therapeutic dose increases drug resistance [11].

Consequently, antibody-conjugated nanoparticles have been investigated and designed to provide targeted delivery of diagnostic and therapeutic agents to the disease sites to overcome some of the challenges. Additionally, antibody-nanoconjugates enable tailored therapeutic administration by carefully controlling their chemistry and minimizing toxicity [12]. Therefore, a greater emphasis is being placed on creating nanotechnology-based therapeutics, termed nanotherapeutics, to improve the diagnosis and treatment of osteosarcoma. As the application of nanotherapeutics increases and sustains the clinical therapeutic benefits of chemotherapeutics, this may result in fewer adverse effects by enhancing their protection, absorption, tumor penetration, and selective distribution [13]. Furthermore, nanoparticles may be modified to allow the targeted administration and controlled release of therapies, keeping drug concentrations specifically at the tumor site and improving their therapeutic impact. For instance, targeted nanoparticles containing drugs have been utilized to inhibit osteosarcomas, such as DOX, gemcitabine, epirubicin, lenvatinib and sorafenib [14].

In addition, epidermal growth factor (EGF) pathways play a vital role in bone metabolism by keeping cells in an undifferentiated state, and its signaling is critical in limb development. The EGF pathways promote chondrocyte proliferation while suppressing hypertrophy and promoting the bone replacement of cartilage. Additionally, EGF and EGF-like proteins also govern the early stages of endochondral ossification by fine-tuning the proliferation and differentiation of bone tissue cells. Furthermore, EGF has been found to be overexpressed in many types of cancers, including osteosarcoma [15]. Moreover, epidermal growth factor receptor (EGFR) is associated with cancer because tyrosine kinase activity is required for tumor survival, growth, and metastasis. Additionally, EGFR promotes epithelial-mesenchymal transition (EMT) and contributes to cancer stemness maintenance. It is also implicated in both primary malignant tumors and metastasis development in bone. EGF stimulation of the MAPK/ERK and PI3K/Akt pathways in osteosarcoma results in cytoskeleton rearrangement, promoting cell proliferation and migration [16]. Moreover, EGFR expression in osteosarcoma cells is associated with poor prognosis and local recurrence and metastasis [17].

Furthermore, radionuclide treatment utilizes radiopharmaceuticals, termed radiopharmaceutical therapy (RPT), which is evolving as an effective and safe targeted method for cancer treatment that combines a particle-emitting radionuclide with a carrier that enables the radionuclide to be targeted to disease areas [18]. In particular, the primary goal of radionuclide treatment is to maximize the amount of damage to cancer cells while reducing the amount of harm to healthy tissue in the surrounding area. RPT delivers systemic therapy by direct administration of the radiotherapeutic and is used to treat a range of malignancies, including thyroid cancer, prostate cancer, neuroendocrine tumors, and bone and liver metastases. However, size limitation is an important design consideration as pore sizes up to 170 nm diameter have been reported in bone marrow fenestrated capillaries [14]. In fact, Sou et al. studied the targeting ability of technetium radiolabeled liposomes with ~216 nm diameter and a negative surface charge to bone marrow in rhesus monkeys; their results determined ~70% uptake by the bone marrow [19]. Hence, this study considered the EGFR targeted NP with a ~175 nm diameter capable of entering bone marrow fenestrated capillaries.

The primary challenge in nuclear medicine is the lack of radiopharmaceuticals that can specifically target certain organs or infected cells in the human body. To address this issue, site-specific radiopharmaceuticals having a stronger affinity for certain organs, for example iodine isotopes for thyroid illnesses such as thyroid cancer, are necessary. In February 2022, Samarium-153−DOTMP (CycloSam), a bone-targeting radiopharmaceutical, was granted a rare pediatric disease status by the U.S. Food and Drug Administration (FDA) to treat osteosarcoma. Samarium-153 has a low specific activity, and the chelator DOTMP eradicates off-target migration and targets high bone turnover sites. Additionally, Radium-223 dichloride is a targeted alpha-particle-emitting treatment approved by the FDA to treat castration-resistant prostate cancer with symptomatic bone metastases. Furthermore, the application of diagnostic and therapeutic radioligands targeting a molecular target provides targeted therapy and prediction of treatment response [20]. Combining radionuclides with on-target antibodies provides an enhanced radiopharmaceutical binding capability. 

Therefore, this study realized the importance of a targeted radiolabeled nanocarrier for treating osteosarcoma bone cancer to overcome some of the significant treatment challenges. We expect the nanotherapeutic developed in this study to preserve the entrapped Na^131^I and DOX from degradation, bind selectively to MG-63 osteosarcoma cells, and minimize Na^131^I and DOX-induced off-target cytotoxicity [21]. In doing so, this will improve the efficacy of osteosarcoma treatment by providing a multiple therapeutic delivery platform. Therefore, we synthesized PEG-PLGA nanoparticles with DOX and Na^131^I encapsulated in the inner core and labeled them with an EGFR antibody to target human MG-63 cells in a three-dimensional (3D) spheroid model. This proof of concept study utilized a 3D model to simulate the three-dimensional physical environment in which osteosarcoma cells develop and interact with the tumor microenvironment (TME), including its extracellular matrix. Furthermore, the nanoparticles were characterized for particle size, zeta potential, polydispersity index, radiochemical purity, and stability. Additionally, we evaluated the pharmacological properties of DOX-loaded PEG-PLGA Na^131^I nanoparticles (DIE-NPs), including cellular absorption, in vitro cytotoxicity, and cell apoptosis. The specific binding of the DIE-NPs to osteosarcoma cells and their ability to induce apoptosis indicates that the multi-therapeutic nanotherapeutic has the potential to treat osteosarcoma.

## 2. Materials and Methods

### 2.1. Materials and Cell Culture

MG-63 osteosarcoma cells (EGFR+) and fibroblast (EGFR−) were provided by the Biomedical Engineering, Faculty of Medicine, Prince of Songkla University. The Na^131^I was procured from the Thailand Institute of Nuclear Technology. Instant thin layer chromatography paper (Medium ITLC-SG) was supplied by Global Medical Solutions, Bangkok, Thailand. CRC-77tHR Dose Calibrator was purchased from Capintec, Inc., NJ, USA. Nuclear staining dye 4′,6-diamidino-2-phenylindole (DAPI) and FITC (fluorescence isothiocyanide) dye were purchased from Thermo Fisher Scientific (Waltham, MA, USA). The human EGFR Alexa Fluor 647-conjugated antibody was purchased from Biolegend. Carboxy-terminated 50:50 PLGA polymer 0.66 dL/g was supplied by LACTEL Absorbable Polymers (Birmingham, AL, USA). The 1,2-distearoyl-sn-glycero-3-phosphoethanolamine-N-[carboxy(polyethylene glycol)-2000] (sodium salt) (DSPE−PEG(2000) Carboxylic Acid) was purchased from Avanti Polar Lipids (Birmingham, AL, USA). Dulbecco’s Modified Eagle Medium (DMEM), phosphate buffer solution (PBS), and chloroform-methanol (0.25% KCl) solution were procured from Thermo Fisher Scientific (Waltham, MA, USA). Bicinchoninic acid (BCA) assay kit and paraformaldehyde were supplied by Millipore Sigma (St. Louis, MO, USA). All purified deionized (DI) water was provided by a Direct-Q3 water purification system. Live/Dead™ cell imaging kit (488/570) was supplied by Thermo Fisher Scientific (Waltham, MA, USA). MTT assay kit (cell proliferation) (colorimetric) was procured from Abcam (Cambridge, MA, USA). All solvents used in the research were procured from Millipore Sigma (St. Louis, MO, USA) and Thermo Fisher Scientific (Waltham, MA, USA). 

### 2.2. Preparation and Characterization of Nanoparticles

**Figure 1:**
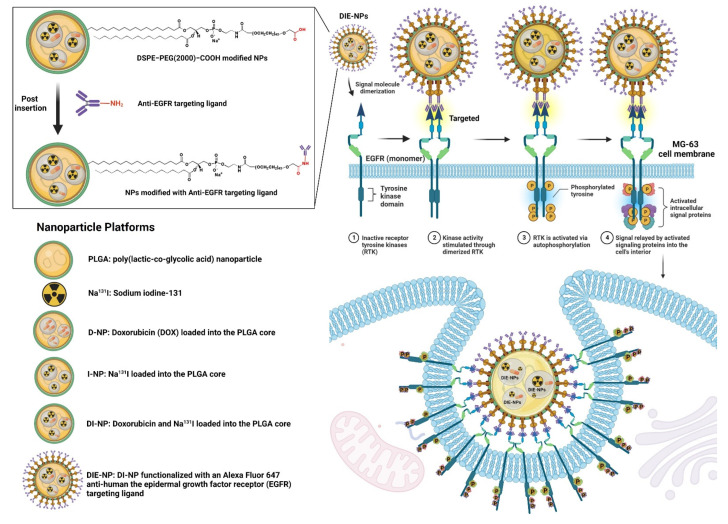
Schematic of DIE-NP nanotherapeutic platform. The fabrication of DSPE−PEG(2000)−COOH modified DIE-NP functionalized with an Alexa Fluor 647 anti-human the epidermal growth factor receptor (EGFR) targeting ligand.

In summary, the synthesis of the various nanoparticles (Figure 1) was as follows:

- Control: Dulbecco’s Modified Eagle Medium (DMEM) cell culture media.
- PLGA: PLGA nanoparticles were synthesized by dissolving the pellets in dichloromethane organic solvent (DCM), and adding FITC (fluorescence isothiocyanate) dye. This was added dropwise to an aqueous solution of 1 × PBS, and agitated gently for 4 h to aid evaporation in a fume hood.
- Na^131^I: sodium iodide (Na^131^I) with an activity of 3.70 MBq (100 µCi), dissolved in 1 × PBS.
- D-NPs: doxorubicin (DOX) loaded into the PLGA cores, prepared by the double emulsion process.
- I-NPs: Na^131^I (activity of 3.70 MBq) loaded into the PLGA cores, prepared by the double emulsion process. Briefly, for the inner phase Na^131^I was diffused with 25 μL of 500 mM 1 × PBS at pH 8, and sonicated for 2 min at 70% pulsed power (2 s on/1 s off) with 500 μL of PLGA in 10 mg/mL dichloromethane (DCM). The solution was then added to 5 mL of 10 mM 1 × PBS at pH 8 and sonicated for 2 min. Then 10 mL of 10 mM 1 × PBS at pH 8 was added to assist evaporation and stirred for 4 h in a fume hood.
- DI-NPs: DOX and Na^131^I (activity of 3.70 MBq) were loaded into the PLGA cores prepared by double emulsion as described in D-NPs and I-NPs (above).
- DIE-NPs: To fabricate the targeted DIE-NPs, the DI-NPs were functionalized with an Alexa Fluor 647 anti-human EGFR antibody targeting ligand. 500 µL of DI-NPs (10 mg/mL) were suspended in 4 mL 1 × PBS, incubated in 0.5 mg DSPE−PEG(2000) carboxylic acid and mixed at 400× *g* for 60 min. They were then rinsed twice with 1 × PBS and mixed for 5 min at 1000× *g*, and suspended in 1 × PBS. Afterward, 1 µg of Alexa Fluor 647 anti-human EGFR antibody targeting ligand was added and stirred gently for 120 min at 4 °C. Lastly, the DIE-NPs were washed with 1 × PBS twice and stored at 4 °C.

Furthermore, dynamic light scattering (DLS) measurements (Malvern ZEN 3600 Zetasizer) were used to evaluate the DIE-NPs physiochemical properties, size, polydispersity and zeta potential at room temperature in triplicate. Additionally, Alexa Fluor 647 was used to verify the binding of the anti-human EGFR antibody to the Na^131^I radiolabeled PLGA NPs and MG-63 (EGFR+) uptake. A fluorescent live cell imaging microscope (LionHeart FX Automated Microscope, BioTek Instrument, Winooski, VT, USA), measured the fluorescence intensity. 

### 2.3. Structural Characterization and Stability of Nanoparticles

Zeta potential measurement was performed to evaluate the conjugation of DSPE−PEG(2000)−COOH to the nanoparticle at room temperature in triplicate. Next, the samples were incubated in 1 × PBS at intervals of 3, 24, 48, 72, and 96 h to evaluate DIE-NPs stability. 

Additionally, DIE-NPs morphology was studied by transmission electron microscopy (TEM) with a JEOL JEM-2010 by applying 1 mg/mL of the DIE-NPs to a TEM glow-discharged carbon-coated grid washed for five minutes with distilled water and stained with 1% uranyl acetate. The grid was dried and photographed using TEM at 200 kV.

Protein quantification determines the amount of Alexa Fluor 647 anti-human EGFR antibody conjugated to the surface of the DIE-NPs. The DIE-NPs conjugates were centrifuged (13,000 rpm for 30 min), and the supernatant containing free anti-human EGFR antibody was analyzed. The protein content in the supernatant was determined using excitation/emission at 633/668 nm in an ImageStreamX Mk II flow cytometer.

### 2.4. Quantification of Ligand Surface Coverage

To functionalize the DSPE−PEG(2000)−COOH (C_131_H_257_NNaO_55_P) with a composition of C 56.59%, H 9.32%, N 0.50%, Na 0.83%, O 31.65%, P 1.11% and a molecular weight of 2780 g/mol with an anti-human EGFR antibody, the DSPE−PEG(2000)−COOH was added to the PLGA solution. A nanoparticle tracking analyzer (NTA) (NanoSight, Malvern analytical) evaluated the coverage and verified that 1 mg of PLGA contained ~8 × 10^9^ nanoparticles. In addition, various quantities of 25, 50, 100, 250, 500, and 1000 mg of DSPE−PEG(2000)−COOH were added to evaluate the ligand surface density of 1 mg of PLGA. To assess the amount of Alexa Fluor 647-labeled anti-human EGFR antibody associated with the nanoparticle, the nanoparticles were hydrolyzed in 1 N NaOH (5 mg/mL) to liberate the FITC-labeled DSPE−PEG−anti-EGFR. After incubation for 1 h, the amount of Alexa Fluor 647-labeled anti-human EGFR antibody was measured by spectrofluorescence (excitation/emission: 633/668 nm), based on a spherical nanoparticle density of 1.2 g/cm^3^ to calculate the ligand density per nanoparticle [22].

### 2.5. Sodium Iodine-131 Radiochemical Purity and Radioactive Stability of DIE-NPs

Additionally, to establish the radiochemical purity of Na^131^I, a fast instant thin-layer chromatography system with a solvent system and three-paper chromatography strip was employed to determine the radiochemical purity standard radiochemical purity percentage (%RCP). Drop by drop, 100 µL of DIE-NPs was applied to a Medium Instant Thin Layer Chromatography (ITLC-SG) plate. The ITLC-SG paper was made using 0.25% KCl solution in chloroform-methanol. Finally, we used a dose calibrator in which only the unbound Na^131^I migrates to the top of the strip to determine the quantity of unbound Na^131^I and DIE-NPs. Additionally, it is a requirement that contaminants should not exceed 5% of total activity [23]. The following formula is used to determine the percent radiochemical purity of DIE-NPs:

(1) $$\%\mathrm{RCP}\;=\frac{\mathrm{Total}\;\mathrm{counts}\;\mathrm{of}\;\mathrm{sample}}{\mathrm{Total}\;\mathrm{counts}\;\mathrm{of}\;\mathrm{sample}\;+\;\mathrm{unbound}\;\mathrm{NaI}}\times 100$$

The DIE-NPs were incubated at 37 °C with 20% fetal bovine serum and 1 × PBS for 0, 1, 3, 6, 12, and 24 h. ITLC was performed on five serial serum samples using an eluent of 0.9% NaCl solution, to evaluate the radiolabeled NPs stability. Over the 24 h, the percentage change in I-131-labeled nanoparticle radiochemical purity was measured in triplicate to determine their stability.

### 2.6. Radioactive Labeling Yield

**Figure 2:**
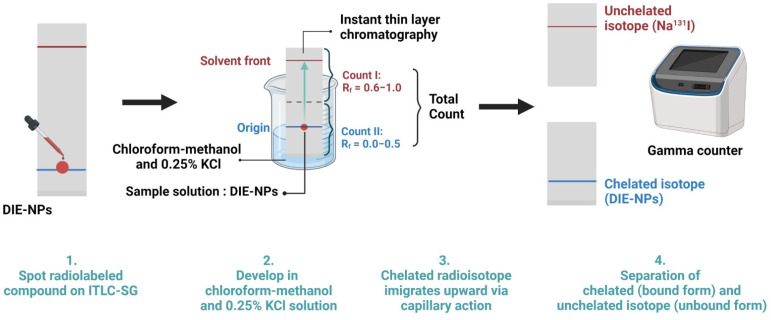
Radioactive labeling yield schematic. Instant thin-layer chromatography (ITLC) silica gel was utilized in an ITLC system with a solvent system. A paper chromatography strip was employed to determine the radioactive labeling yield after labeling and subsequently developed in chloroform-methanol and 0.25% KCl solution. Analyzes of bound and unbound forms were measured using a dosage calibrator.

Instant thin-layer chromatography (ITLC) silica gel was used with 100 µL aliquots on a fast ITLC system with a solvent system, and a three-paper chromatography strip was employed to determine the radioactive labeling yield. After labeling for 0, 1, 3, 6, 12 and 24 h and subsequently development in chloroform-methanol and 0.25% KCl solution, analyses were performed using a dosage calibrator in which only the unbound Na^131^I migrates to the top of the strip. Radioactive spots were detected using their retention factor (R_f_) values. Na^131^I that is unbound migrates with the solvent between R_f_ = 0.6 to 1.0, while DIE-NPs migrate at R_f_ = 0.0 (Figure 2).

### 2.7. Calculation of Na^131^I Radiation Dose Delivered to Each Well

The radiation dosage per well of Na^131^I activity 3.70 MBq (100 µCi) was calculated by the formula [23]:

(2) $$D\;=\;Ã\;\times \frac{\Delta \beta }{100}$$

where D is the absorbed Na^131^I radiation dose, Ã the cumulative Na^131^I activity, and Δβ is the β rays absorbed dose constant.

(3) $$Ã\;=\overset{t}{\underset{0}{\int }}A\left(t\right)\;d\left(t\right)$$

(4) $$A\left(t\right)={\;A}_{0}{\;e}^{-\lambda t}$$

(5) $$Ã\;=\frac{A_{0}\left[\;\left(1-e^{-\lambda t}\right)\right]}{\lambda }\;$$

where A(t) is the activity at time t, A_0_ is the initial Na^131^I activity at t = 0, and λ is the decay. 

### 2.8. The Two-Dimensional (2D) In Vitro Therapeutic I-131 Dose Optimization

An MTT assay was used to analyze the cell proliferation rate to determine the in vitro treatment response. Dulbecco’s Modified Eagle Medium DMEM supplement containing 1% penicillin/streptomycin, 10% fetal bovine albumin, and L-glutamine (Gibco-BRL) was used to cultivate human MG-63 osteosarcoma cells in a humidified incubator at 37 °C and 5% CO_2_ and cultivate them to 60–80% confluency. Then 5000 MG-63 cells per well were plated into 96-well microtiter plates at 200 µL/well, 24 h before treatment. They were treated with DIE-NPs (*n* = 3) for 24 h, at doses of 0, 0.37, 1.85, 3.70, 5.55 and 7.40 MBq. The DIE-NPs were then rinsed before being cultivated in fresh culture at a final 200 µL/well volume and stirred for 24 h, followed by MTT assessment to evaluate the MG-63 cell proliferation rate after treatment. 50 µL of MTT solution was incubated with 50 µL of serum-free medium at 5% CO_2_ and 37 °C for 3 h in 96-well microtiter plates. Then, 150 µL MTT solvent was added to each well before shielding the well plates with aluminum foil to avoid light ingress and agitated for 15 min on an orbital shaker. The treated cells absorbance was then evaluated by a microplate reader (optical density = 590 nm), and the intensity was relative to the quantity of viable MG-63 cells. The cell proliferation rate percentage (P%) calculation for each dosage is as follows:

(6) $$P\;\left(\%\right)=\frac{\mathrm{ODc}\;-\;\mathrm{ODs}}{\mathrm{ODc}}\times 100$$

where P (%) is the cell proliferation rate, ODc is the control mean optical density, and ODs the mean optical density. The proliferation rate percentage is the mean of three measures and specified as a percentage. In addition, the greater the metabolic activity of MG-63 cells, the higher the signal detected, indicating the degree of MG-63 cell proliferation, cytotoxicity, and cell viability.

### 2.9. Radioactive Encapsulation Efficiency

The DIE-NPs radioactive encapsulation efficiency was assessed by extracting 3.70 MBq using 0.1 M HCl in acetonitrile solution at time points 0, 3, 6, 12 and 24 h, and the I-131 activity was measured with a gamma counter. The encapsulation efficiency was calculated by Equations (5) and (7) for the radioactive decay at 0, 3, 6, 12 and 24 h, and performed in triplicate. The average of the three results (mean ± standard deviation; *n* = 3) is presented as the DIE-NPs encapsulation efficiency.

(7) $$\mathrm{Radioactive}\;\mathrm{encapsulation}\;\mathrm{efficiency}\;\left(\%\right)=\frac{\mathrm{total}\;\mathrm{Iodine}\;131\;\mathrm{activity}\;\mathrm{added}\;-\;\mathrm{not}\;\mathrm{encapsulated}\;\mathrm{Iodine}\;131}{\mathrm{total}\;\mathrm{Iodine}\;131\;\mathrm{activity}\;\mathrm{added}}\times 100$$

### 2.10. Two-Dimensional (2D) In Vitro Therapeutic DOX Optimization

In vitro DOX optimization was determined using the MTT assay to determine the cell proliferation rate. MG-63 (EGFR+) were cultured in Dulbecco’s Modified Eagle Medium (DMEM) supplemented with 1% penicillin/streptomycin, 10% fetal bovine albumin, and L-glutamine (Gibco-BRL). MG-63 cells were cultivated to 60–80% confluency in culture flasks at 37 °C and 5% CO_2_ in a humidified incubator. 24 h before treatment, 5000 cells per well were plated onto 96-well microtiter plates with a final volume of 200 μL for each well. The MG-63 cells were then treated with free DOX and DIE-NPs (*n* = 3) at DOX concentration of 5 × 10^−5^, 1 × 10^−4^, 5 × 10^−4^, 1 × 10^−3^, 5 × 10^−3^, 1 × 10^−2^, 5 × 10^−2^, 1 × 10^−1^, and 5 × 10^−1^ µg/mL for 24 h. The free DOX or DIE-NPs was washed and cultivated in the new culture mix for 24 h at a final volume of 200 µL/well. Following that, the cell proliferation rate was determined using the MTT test, incubating 50 μL serum-free medium with 50 µL MTT solution in 96-well microtiter plates for 3 h at 37 °C and 5% CO_2_. After adding 150 µL MTT solvent to each well, the well plates were covered with aluminum foil to avoid light penetration and stirred for 15 min in an orbital shaker. The microplate reader set to optical density (OD) = 590 nm was used to determine the absorbance of the treated cells. The color intensity generated was related to the number of viable cells.

### 2.11. Drug Loading and Encapsulation Efficiency

By changing the DOX initial input the DOX drug loading into the PLGA NP cores can be regulated. Amounts of 5, 10, 15, and 20 wt% (DOX weight/PLGA weight) were used, and the DOX-loaded NPs with a range of initial drug inputs were measured for size. The drug loading yield and drug encapsulation efficiency with a range of initial DOX inputs were assessed by determining their fluorescence with a microplate reader (480 nm excitation; 580 nm emission). Drug loading and encapsulation efficiency calculated by Equations (8) and (9):

(8) $$\mathrm{Drug}\;\mathrm{loading}\;\left(\%\right)=\frac{\mathrm{Weight}\;\mathrm{of}\;\mathrm{the}\;\mathrm{total}\;\mathrm{DOX}\;\mathrm{added}\;-\;\mathrm{Weight}\;\mathrm{of}\;\mathrm{the}\;\mathrm{free}\;\mathrm{unentrapped}\;\mathrm{DOX}}{\mathrm{Weight}\;\mathrm{of}\;\mathrm{DOX}\;\mathrm{loaded}\;\mathrm{NPs}}\times 100$$

(9) $$\mathrm{DOX}\;\mathrm{Encapsulation}\;\mathrm{efficiency}\;\left(\%\right)=\frac{\mathrm{Weight}\;\mathrm{of}\;\mathrm{the}\;\mathrm{total}\;\mathrm{DOX}\;\mathrm{added}\;-\;\mathrm{Weight}\;\mathrm{of}\;\mathrm{the}\;\mathrm{free}\;\mathrm{unentrapped}\;\mathrm{DOX}}{\mathrm{Weight}\;\mathrm{of}\;\mathrm{the}\;\mathrm{total}\;\mathrm{DOX}\;\mathrm{added}}\times 100$$

### 2.12. Cumulative Amount of Drug Release

A DOX cumulative drug release investigation was conducted using Slide-A-Lyzer MINI Dialysis Cups (Sigma-Aldrich, St. Louis, MO, USA), by dialyzing DOX samples against 1 × PBS, with a molecular weight cutoff of 3.5 kDa. The dialysis cups were placed in a beaker containing 2000 mL of 1 × PBS buffer (pH = 7.4) release medium, and agitated at 200 rpm and 37 °C to maintain a consistent pH and sink state. Next, the samples were collected from the buffered medium solution at time intervals of 0, 0.5, 1, 3, 6, 12, 24, 48, 72, 96 and 120 h and replaced with the same volume of new buffer medium. Then DOX in the NPs was removed using a solution of 0.1 M HCl in acetonitrile, and the fluorescence was measured to calculate the DOX concentration (480 nm excitation; 580 nm emission). All experiments were performed in triplicate, and values are presented as the mean of the three values (mean ± standard deviation; *n* = 3). The cumulative amount of drug release was calculated by the following Equation (10):

(10) $$\mathrm{Cumulative}\;\mathrm{amount}\;\mathrm{of}\;\mathrm{DOX}\;\mathrm{release}\;\left(\%\right)=\frac{V_{e}+\sum_{1}^{n-1}\left(C_{i}+V_{0}C_{n}\right)}{m_{0}}\times 100$$

where *V_e_* is the volume of release media removed at time intervals, *V*_0_ is the total volume of release medium, *C_i_* is the concentration of DOX in the release medium, and *m*_0_ is the total mass of DOX entrapped in nanoparticles.

### 2.13. Three-Dimensional (3D) Human MG-63 Tumor Spheroid In Vitro Cytotoxicity

To assess the cell viability of 5000 and 10,000 initial MG-63 cell spheroids, a 3D Cell Titer-Glo^®^ cell viability assay was conducted after 72 h treatment with control, PLGA, Na^131^I, D-NPs, I-NPs, DI-NPs, and DIE-NPs at an I-131 equivalent activity of 3.70 MBq (100 µCi). The medium was removed as indicated by the manufacturer, and 100 µL of Cell Titer-Glo^®^ reagent was added to every well, blended for 2 min, followed by incubation for 30 min at room temperature. The luminescence signal was then measured using a plate reader (Tecan, Infinite 200 PRO). Additionally, the morphology of 5000 and 10,000 initial MG-63 cell spheroids was imaged by live cell imaging microscope (bright-field channel).

### 2.14. In Vitro Surface Immunofluorescence Cellular Binding/Uptake Targeting Efficiency 

The MG-63 (EGFR+) and fibroblast (EGFR−) cells intracellular binding/uptake (*n* = 3) was ascertained by incubating the cells for 24 h with DIE-NPs at an activity of 3.70 MBq at 200 µL/well final volumes. Next, the MG-63 and fibroblast cells were rinsed with 1 × PBS, and fixed with 4% paraformaldehyde for 20 min to conserve the samples before being washed with 1 × PBS. They were then stained with 4′,6-diamidino-2-phenylindole (DAPI) dye for 30 min at 37 °C before being rinsed twice before fluorescent live cell imaging microscope examination.

ImageStreamX Mk II was used for the nanoparticle surface immunofluorescence and cellular binding/uptake flow cytometer. The MG-63 cells were grown to 80% confluency at 37 °C with 5% CO_2_ in a humidified incubator. Next, the cells were detached from the culture flasks with 0.25% Trypsin-EDTA solution. After centrifuging the cells at 4000 rpm for 3 min, 10^6^ cells were collected, and the supernatant was washed twice with 1 × PBS at 4 °C. Then, FITC green fluorescent dye was encapsulated inside the PLGA, Na^131^I, D-NPs, I-NPs, DI-NPs, and DIE-NPs prior to treatments. Following this, the MG-63 single cell suspensions were treated (*n* = 3) with either a control treatment, PLGA, Na^131^I, D-NPs, I-NPs, DI-NPs or DIE-NPs (activity 3.70 MBq) for 1 h. The cells were then rinsed with 2 mL of 1 × PBS, centrifuged at 350× *g* for 5 min, and then washed once more. The MG-63 cells were then suspended in 1 × PBS buffer and analyzed using a flow cytometer. Cellular fluorescence was measured using a flow cytometer in bright-field (gray channel), FITC (green channel), and Alexa Fluor 647 (red channel) for 5 × 10^4^ cells/sample.

### 2.15. Human MG-63 Tumor Spheroid Three-Dimensional (3D) In Vitro Live/Dead Cell Imaging

Following DIE-NPs treatment, the MG-63 cells were grown in DMEM media supplemented with 1% penicillin/streptomycin, 1% L-glutamine and 10% fetal bovine albumin, and were imaged by a two-color fluorescent live/dead cell imaging kit (488/570) (ThermoFisher Scientific, R37601). The MG-63 spheroids were synthesized as described by Zhang et al. [24]. The MG-63 cells were then cultured at 37 °C with 5% CO_2_ in a humidified incubator to 60–80% confluency. Then, for 72 h previous to treatment, 5 × 10^3^ MG-63 cells/well were seeded on 96-well ultra-low attachment plates with a final volume of 200 µL/well. Afterward, the MG-63 spheroids were treated for 72 h with untreated (control), PLGA, Na^131^I (activity 3.70 MBq), D-NPs, I-NPs (activity 3.70 MBq), DI-NPs (activity 3.70 MBq), and DIE-NPs (activity 3.70 MBq). This was replicated three times. The manufacturer’s instructions were then followed to create a two-color fluorescent live/dead Cell Imaging test. First, Live Green (Comp. A) was mixed with Dead Red (Comp. B) to create a 2 × working solution. After pouring 50 µL of 2 × working solution into each well and incubating at 25 °C for 15 min, LionHeart live cell imaging was applied to image the cells. 

In addition, to assess the cell viability of MG-63 spheroids, a 3D Cell Titer-Glo^®^ cell viability assay was conducted after 72 h treatment with control, PLGA, Na^131^I, D-NPs, I-NPs, DI-NPs, and DIE-NPs at an I-131 equivalent activity of 3.70 MBq (100 µCi). The medium was removed as indicated by the manufacturer, and 100 µL of Cell Titer-Glo^®^ reagent was added to every well, with blending for 2 min, and 30 min incubation at room temperature. The luminescence signal was then measured using a plate reader (Tecan, Infinite 200 PRO).

### 2.16. Three-Dimensional (3D) Human MG-63 Tumor Spheroid DIE-NPs Penetration 

The penetration of the DIE-NPs into the spheroids was investigated using a ~500 µm diameter 3D spherical MG-63 human spheroid model. The spheroids were treated for 24, 48, and 72 h with one spheroid per well by DIE-NPs with an activity of 3.70 MBq (100 µCi). The spheroids were washed and rinsed with 1 × PBS before being fixed for 60 min in 1 × PBS containing 4% paraformaldehyde. The spheroids were then rinsed and stained with DAPI dye before LionHeart live cell imaging scanning and their fluorescence intensity measured.

### 2.17. Cell Cycle

For at least 24 h, MG-63 cells were cultured at 37 °C with 5% CO_2_ in an incubator after being seeded at 10^5^–10^6^ cells/well in DMEM supplemented media. After 48 h of incubation, the cells were treated with control, PLGA, Na^131^I, D-NPs, I-NPs, DI-NPs, and DIE-NPs. Once the medium was removed, the cells were rinsed with 1 × PBS and harvested using trypsin/EDTA). Then the trypsinized cells were rinsed with an assay buffer, and the ensuing cell pellets were suspended at a density of 1 × 10^6^ cells/mL in fresh assay solution. Next, the cells were fixed and permeabilized for at least 2 h with 1 mL of a fixative agent before staining with propidium iodide (PI). Afterward, the cells were centrifuged at 500× *g* for 5 min, and the fixative was wholly removed. Cell cycle analysis was then performed using ImageStreamX Mk II flow cytometer after the cell pellets were suspended in the PI staining solution and incubated for 30 min at room temperature in the dark.

### 2.18. Blood Compatibility of Samples: Hemolysis Assay

To inhibit the formation of blood clots, the plasma was extracted from human blood in the presence of heparin anticoagulant. RBCs were suspended at 8 × 10^9^ cells/mL in positive control (DMSO), Na^131^I (activity 3.70 MBq), D-NPs, I-NPs (activity 3.70 MBq), DI-NPs (activity 3.70 MBq), and DIE-NPs (activity 3.70 MBq) and centrifuged for 5 min at 500× *g*. At 0, 3, 6, 12, 24, 36, 48, and 80 h, the supernatants were collected at 37 °C to test blood lysis. To calculate the hemolysis (%), a BCA protein assay kit was used to evaluate the protein in the samples before measuring the hemoglobin absorbance using a microplate reader (OD = 562 nm).

### 2.19. Cellular Uptake by Macrophage Cells

The macrophage cells (RAW 264.7 cells) intracellular binding/uptake (*n* = 3) was demonstrated for non-coated bare PLGA nanoparticles (Bare NPs) and DIE-NPs in comparison to PEGylated nanoparticles (PEG-NPs) by incubating the cells with DIE-NPs at an activity of 3.70 MBq at 200 µL/well final volumes. Next, the macrophage cells were rinsed with 1 × PBS, and fixed with 4% paraformaldehyde for 20 min to conserve the samples before being washed with 1 × PBS. They were then stained with 4’,6-diamidino-2-phenylindole (DAPI) dye for 30 min at 37 °C before being rinsed twice and examination using a fluorescent live cell imaging microscope.

### 2.20. Statistical Analysis

All study studies were carried out in triplicate (analytically and experimentally), and all results and the data reported as mean ± standard deviation. GraphPad Prism 8.0 was used to perform all statistical analyses (GraphPad Software Inc., La Jolla, CA, USA). The unpaired, two-tailed Student’s *t*-test used to determine any statistically significant difference between two groups. Furthermore, one-way ANOVA was used to compare the means of two or more independent groups to see whether they were significantly different, with *p* < 0.05 indicating statistical significance. 

## 3. Results

### 3.1. Nanoparticle Physicochemical Properties and Characterization

**Figure 3:**
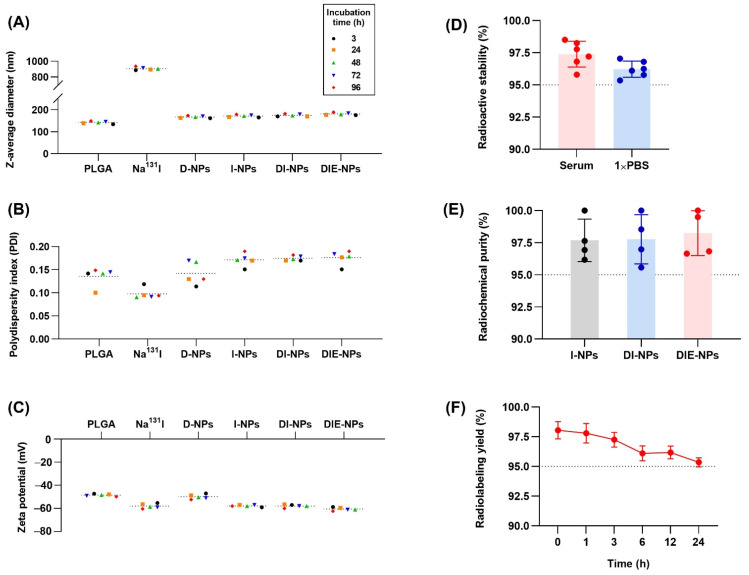
Physiochemical properties and characterization of the nanoparticles after incubation times of 3, 24, 48, 72 and 96 h. (**A**) Z-average diameter (nm). (**B**) Polydispersity index. (**C**) Zeta potential (mV). (**D**) Radioactive stability (%) of DIE-NPs incubated in 20% fetal bovine serum and 1 × PBS over 24 h. (**E**) Radiochemical purity (%) of I-NPs, DI-NPs and DIE-NPs over 96 h. (**F**) Radiolabeling yield of DIE-NPs over 24 h. Data are given as mean ± standard deviation (*n* = 3).

Dynamic light scattering (DLS) was performed and determined that the z-average diameter (nm) of Na^131^I was ~887 nm, DOX loaded NPs (D-NPs) ~161 nm, Na^131^I radiolabeled NP (I-NPs) ~165 nm, DOX loaded Na^131^I radiolabeled NPs (DI-NPs) ~170 nm, and the anti-EGFR DOX loaded Na^131^I radiolabeled NPs (DIE-NPs) ~175 nm (Figure 3A). In addition, the polydispersity index (PDI), indicating the uniformity of the nanoparticle distribution, revealed that the DIE-NPs have a PDI of 0.15 (Figure 3B). Furthermore, the zeta potential (mV) (Figure 3C), measured by DLS, indicated the DIE-NPs had a zeta potential of −59 mV, the DI-NPs a zeta potential of −57 mV and the I-NPs −59 mV. Additionally, the radioactive stability illustrated in Figure 3D shows the DIE-NPs having good stability over 24 h, with both the DIE-NPs in serum and 1 × PBS having a stability greater than 95% over a period of 24 h. Furthermore, the DIE-NPs have a radiochemical purity >96% over 96 h (Figure 3E), and a radiolabeling yield >95% over 24 h (Figure 3F). Radioactive stability, radiochemical purity and radiolabeling yield should not be less than 95% under the World Health Organization Consultation Document standard [25].

### 3.2. Structural Characterization and Stability of Nanoparticles

**Figure 4:**
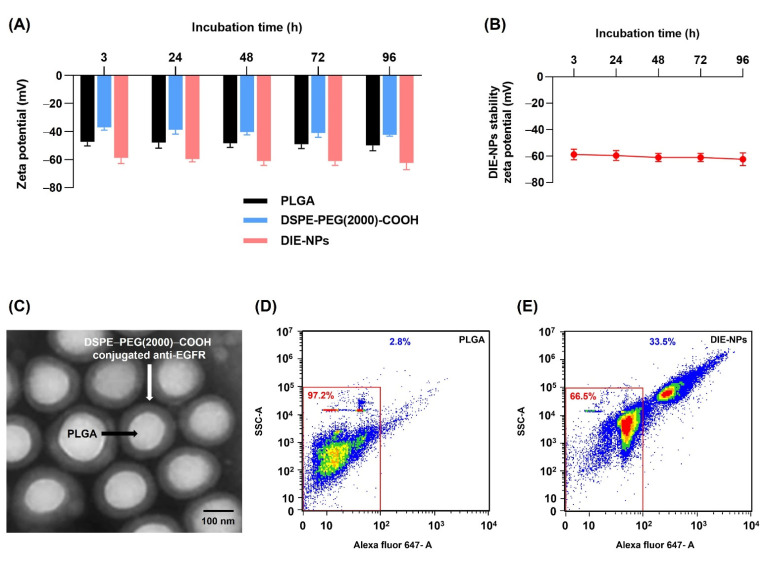
Nanoparticle structural characterization and stability. (**A**) Zeta potential of PLGA, DSPE−PEG(2000)−COOH and DIE-NPs after incubation times of 3, 24, 48, 72 and 96 h. (**B**) DIE-NPs stability zeta potential of DIE-NPs after incubation times of 3, 24, 48, 72 and 96 h. (**C**) Transmission electron microscopy (TEM) image of the DIE-NPs core–shell structure. Scale bar = 100 nm. (**D**) Scatter plot of unconjugated PLGA nanoparticles. (**E**) Scatter plot of conjugated DIE-NPs. The region from flow cytometer evaluated (red line) was adjusted to the upper right corner, which indicates the conjugation of Alexa Fluor 647 anti-human EGFR antibody (fluorescently positive events). Data are given as mean ± standard deviation (*n* = 3).

The zeta potential was performed to evaluate the conjugation of DSPE−PEG(2000) −COOH to the nanoparticle in the 1 × PBS solution at intervals of 3, 24, 48, 72, and 96 h and evaluate DIE-NPs stability (Figure 4A,B). 

The TEM image (Figure 4C) shows the DIE-NPs are comprised of a hydrophobic poly (lactic-co-glycolic acid) (PLGA) core, and a hydrophilic poly(ethylene glycol) (PEG) shell with DSPE−PEG(2000)−COOH conjugated anti-EGFR.

Figure 4D,E demonstrate flow cytometer results for unconjugated PLGA NPs and DIE-NPs. The region evaluated (red line) was adjusted to the upper right corner, which indicates the presence of conjugates containing Alexa Fluor 647 anti-human EGFR antibody (fluorescently positive events). The large population in the lower left corner is based on non-fluorescent dust/debris observed in flow cytometer results for pure PBS.

### 3.3. Quantification of Ligand Surface Coverage

The nanoparticle surface density of the anti-EGFR ligands was ascertained using spectrofluorimetry, to calculate the Alexa Fluor 647-labeled anti-human EGFR. Verifying 500 µg of DSPE−PEG(2000)−COOH, resulted in ~719 molecules/NP, a surface density of ~180 nmol/mg NP, and 150 nmol/cm^2^ NP surface coverage. Additionally, 1 µg anti-human EGFR antibody resulted in ~1125 molecules/NP, a surface density of 7.46 × 10^−3^ nmol/mg NP, and 6.22 × 10^−3^ nmol/cm^2^ NP surface coverage. The intensity of the anti-EGFR ligands bonding was proportional to the ligand quantity added to the nanoparticle synthesis (Table 1).

### 3.4. In Vitro Two-Dimensional (2D) Therapeutic I-131 Dose Optimization

**Figure 5:**
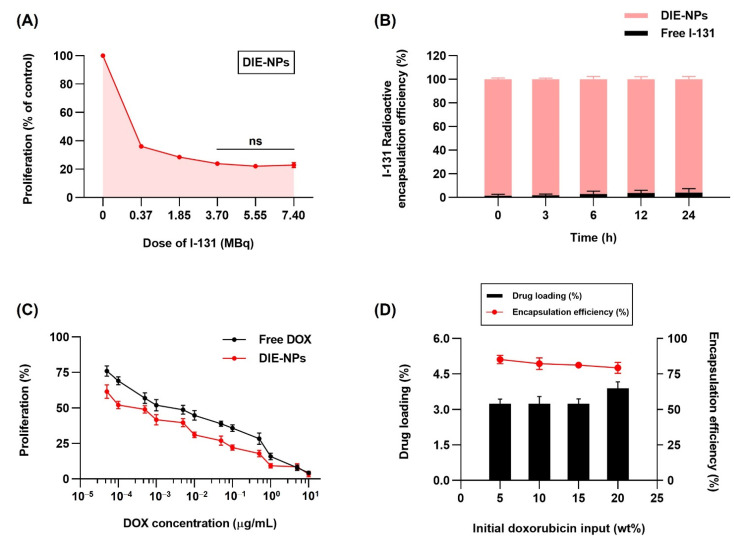
The two-dimensional (2D) in vitro therapeutic optimization. (**A**) MTT assay in vitro cytotoxicity of DIE-NPs at dosages of 0, 0.37, 1.85, 3.70, 5.55, and 7.40 MBq after 24 h incubation. ns = no statistically significant difference in efficacy of destroying cancer cells between 3.70, 5.55, and 7.40 MBq (*p* > 0.05). (**B**) I-131 radioactive encapsulation efficiency (%) of DIE-NPs and free I-131 after 0, 3, 6, 12 and 24 h incubation. (**C**) In vitro cytotoxicity using MTT assay of DIE-NPs and free DOX after 24 h incubation. (**D**) Drug loading and encapsulation efficiency (%) of 5%, 10%, 15%, and 20% initial doxorubicin input (wt%). Data are given as mean ± standard deviation (*n* = 3).

Figure 5A demonstrates the MTT assay evaluation of the cytotoxicity of DIE-NPs at different Na^131^I dosage levels of 0, 0.37, 1.85, 3.70, 5.55, and 7.40 MBq on MG-63 cells. The graph indicates lower proliferation at Na^131^I dosages of 3.70, 5.55, and 7.40 MBq. The DIE-NPs Na^131^I doses of 3.70 MBq, 5.55 MBq, and 7.40 MBq resulted in a proliferation rate of 24%, 22%, and 23%, respectively. Likewise, Hosseinimehr et al. reported that a dose of 3.70 MBq produced an 8.5-fold increase in I-131 genotoxicity on human cultured lymphocytes [26].

### 3.5. Radioactive I-131 Encapsulation Efficiency 

The DIE-NPs, I-131 activity 3.70 MBq encapsulation efficiency at 0, 3, 6, 12 and 24 h is illustrated in Figure 5B. Encapsulation efficiencies of 98.5%, 98.2%, 97.1%, 96.2% and 95.9%, respectively, were obtained, indicating that the DIE-NPs have good stability over a 24 h period.

### 3.6. In Vitro Therapeutic DOX Optimization, Drug Loading and Encapsulation Efficiency of the DIE-NPs

DOX in vitro therapeutic optimization of DIE-NPs and free DOX was assessed by MTT assay after 24 h incubation. MG-63 cells were treated at DOX concentrations of 5 × 10^−5^, 1 × 10^−4^, 5 × 10^−4^, 1 × 10^−3^, 5 × 10^−3^, 1 × 10^−2^, 5 × 10^−2^, 1 × 10^−1^, and 5 × 10^−1^ µg/mL for 24 h (Figure 5C). IC_50_ was achieved with 5 × 10^−4^ µg/mL of DIE-NPs. 

The DOX loading yield and DOX encapsulation efficacy of DIE-NPs were studied to attain the optimal drug formulation. Figure 5D reveals that increasing the DOX initial loading into the PLGA core caused the DOX loading yield to increase. On the other hand, the DOX encapsulation efficacy was reduced. Therefore, it was determined that a 5 wt% DOX loading (5 × 10^−4^ µg/mL) was the optimal formulation, which was used for this study, and achieved an encapsulation efficacy of ~85% and IC_50_. 

### 3.7. Drug Loading and Encapsulation Efficiency of Nanoparticles 

**Figure 6:**
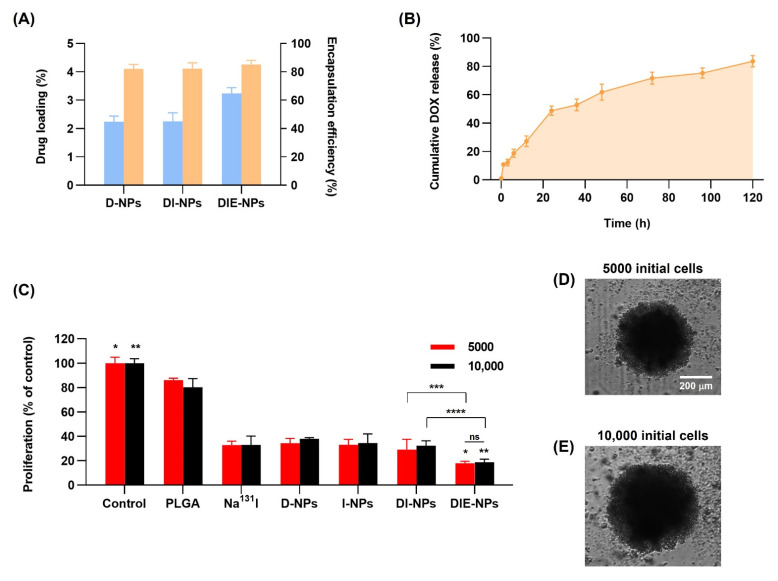
Therapeutic efficiency of nanoparticles. (**A**) Drug loading and encapsulation efficiency of D-NPs, DI-NPs, and DIE-NPs. (**B**) Cumulative amount of DOX release after 0, 1, 3, 6, 12, 24, 36, 48, 72, 96, and 120 h. (**C**) In vitro three-dimensional (3D) MG-63 human tumor spheroid proliferation (% of control) using Cell Titer-Glo^®^ 3D cytotoxicity assay of DIE-NPs compared to control, PLGA, Na^131^I, D-NPs, I-NPs, DI-NPs, and DIE-NPs treatment of 5000 initial cells/well and 10,000 initial cells/well after 72 h incubation. ns = no statistically significant difference in efficacy of proliferation (% of control) between DIE-NPs treatment of 5000 and 10,000 initial cells/well (*p* > 0.05). * denotes significance between control and DIE-NPs of 5000 initial cells/well (*p* < 0.05). ** denotes significance between control and DIE-NPs of 10,000 initial cells/well (*p* < 0.05). *** denotes significance between DI-NPs and DIE-NPs of 5000 initial cells/well (*p* < 0.05). **** denotes significance between DI-NPs and DIE-NPs of 10,000 initial cells/well (*p* < 0.05). (**D,E**) The morphology of three-dimensional (3D) MG-63 human tumor spheroid of 5000 and 10,000 initial cells were imaged in bright-field using LionHeart live cell imaging. [10× images, scale bar = 200 μm]. Data are given as mean ± standard deviation (*n* = 3).

The DOX loading (Figure 6A) illustrates the loading efficiencies of the D-NPs, DI-NPs, and the DIE-NPs, having a drug loading efficiency of 2%, 2%, and 3%, respectively. In addition, to the DOX encapsulation efficiency, it can be observed that the D-NPs, DI-NPs, and the DIE-NPs all have a drug encapsulation efficiency of ~85%.

### 3.8. Cumulative Amount of DOX Release

Figure 6B illustrates an initial burst release of 11% of DOX in the first hour followed by a 3 h subsequent DOX release phase of 12% at 3 h, 19% at 6 h, and 27% at 12 h. This indicates a stable release over 24 h of 49% DOX after the initial release phase. Furthermore, the DIE-NPs cumulative drug release investigated over 5 days, demonstrates a sustained DOX release. Additionally, Figure 6B illustrates that ~53% of the DOX release occurred within the first 36 h, and ~80% of DOX total release, over 5 days, confirming that the majority of the DOX release was within the first 72 h. Furthermore, the DOX release over the 5 days is the result of the DOX drug molecules gradually diffusing through the PLGA NP matrix. This slow diffusion occurrence has been reported previously by Aryal et al. [27].

### 3.9. Three-Dimensional (3D) Human MG-63 Tumor Spheroid In Vitro Cytotoxicity

Cell Titer-Glo^®^ 3D cytotoxicity assay was performed to determine the nanoparticle formulations’ cell cytotoxicity against MG-63 spheroids of ~500 µm. The liquid overlay technique is used to cultivate spheroids in a scaffold-free solution in ultra-low attachment plates, enabling the MG-63 cells to attach to one another under non-adherent three-dimensional culture conditions. This facilitates the division and renewal of MG-63 spheroids because of their infinite proliferation ability. The MG-63 cells were cultured in 5000 cells/well or 10,000 cells/well, and after 72 h treatment, the cell cytotoxicity was analyzed. Treatment of the 5000 cells/well MG-63 spheroids by the non-targeted DI-NPs resulted in ~29% proliferation of control and the targeted DIE-NPs cytotoxicity of ~18%, representing a ~9% increase in cytotoxicity compared to the non-targeted DI-NPs (Figure 6C). In addition, the 10,000 cells/well MG-63 spheroids treated by the non-targeted DI-NPs resulted in ~32% proliferation of control and the targeted DIE-NPs ~19% proliferation of control. This indicates that the targeted DIE-NP has a ~1.7-fold greater cytotoxicity than the non-targeted DI-NPs. Therefore, both the 5000 and the 10,000 cytotoxicity assays confirm the viability of the DIE-NPs specific MG-63 spheroid anti-EGFR targeting. Additionally, the morphology of the three-dimensional (3D) MG-63 human tumor spheroid of 5000 and 10,000 initial cells of ~500 µm-diameter was examined to determine the nanoparticle formulations’ cell cytotoxicity, shown in Figure 6D,E.

### 3.10. EGFR Antibody Cellular Binding/Uptake In Vitro Targeting Efficiency 

**Figure 7:**
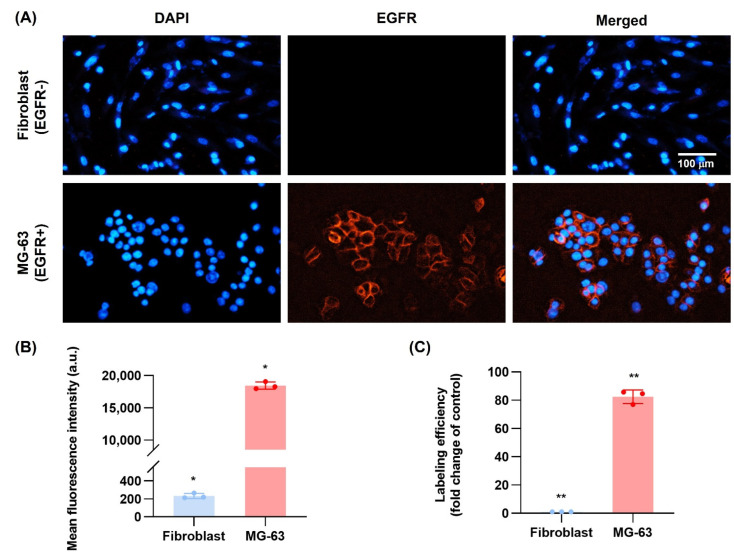
Co-localization of DIE-NPs upon cellular binding/uptake. (**A***)* In vitro EGFR antibody targeting efficiency upon cellular binding/uptake of fibroblast (EGFR−) and MG-63 (EGFR+). DIE-NPs were synthesized using PLGA cores and conjugated with anti-EGFR labeled with Alexa Fluor 647 (red channel). DAPI (blue channel) was used to stain the nucleus. To eliminate out-of-focus fluorescent signals, software was applied to all channels [10× images, scale bar = 100 µm]. (**B**) DIE-NPs mean fluorescence intensity quantification on fibroblast and MG-63 cells. * denotes significance in mean fluorescence intensity of EGFR antibody targeting efficiency upon cellular binding/uptake of DIE-NPs between fibroblasts (EGFR−) and MG-63 (EGFR+) (*p* < 0.05). (**C**) Quantification of the labeling efficiency (fold change of control) of DIE-NPs on fibroblast and MG-63 cells. ** denotes significance in labeling efficiency (fold change of control) of DIE-NPs between fibroblasts (EGFR−) and MG-63 (EGFR+) (*p* < 0.05). Data are given as mean ± standard deviation (*n* = 3).

It is reported that all osteosarcoma tissues and cell lines commonly express EGFR membrane receptors and are associated with osteosarcoma cell differentiation, proliferation and migration [17,28]. This constitutes a potential target for anti-EGFR. Therefore, flow cytometer was used to evaluate the DIE-NPs surface immunofluorescence and MG-63 cellular binding/uptake. The fibroblast (EGFR−) and MG-63 (EGFR+) cells were stained with DAPI fluorescent stain with an affinity for DNA. The DAPI staining (blue channel) can be observed on the nucleus of the MG-63 and fibroblast cells, as illustrated in Figure 7A. Furthermore, EGFR labeling efficacy was visualized by conjugating Alexa Fluor 647 to the anti-EGFR targeting ligands. The fluorescent live-cell imaging analysis verified the cellular binding to cells (red channel). Moreover, the DIE-NPs are highlighted and can be seen binding to the MG-63 cells with a strong mean fluorescent intensity of 18,445 a.u. (Figure 7B) while showing minimal binding to the fibroblast cells with a low mean fluorescent intensity of 213 a.u. As a result, the EGFR targeted DIE-NPs have a ~80-fold greater targeting efficacy to MG-63 cells than fibroblast cells (Figure 7C).

**Figure 8:**
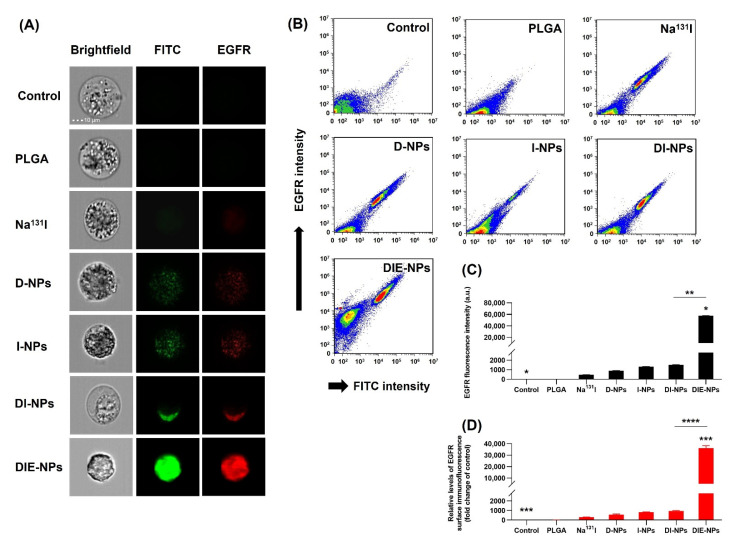
In vitro EGFR antibody cellular binding/uptake targeting efficiency. (**A**) Flow cytometer images of cellular uptake and surface immunofluorescence of MG-63 cells treated with control, PLGA, Na^131^I, D-NPs, I-NPs, DI-NPs and DIE-NPs collected by bright-field (gray channel), Alexa Fluor 647-anti EGFR (red channel) and FITC (green channel). Scale bar = 10 µm. (**B**) Scatter plot of cellular uptake and surface immunofluorescence intensity of MG-63 cells treated with control, PLGA, Na^131^I, D-NPs, I-NPs, DI-NPs and DIE-NPs. (**C**) Quantification of Alexa Fluor 647-anti EGFR (red channel) fluorescence intensities. * denotes significance in fluorescence intensity of EGFR antibody targeting efficiency upon cellular binding/uptake between control and DIE-NPs (*p* < 0.05), and ** denotes significance in fluorescence intensity of EGFR antibody targeting efficiency upon cellular binding/uptake between DI-NPs and DIE-NPs (*p* < 0.05). (**D**) Relative levels of Alexa Fluor 647-anti EGFR surface immunofluorescence (fold change of control). *** denotes significance in relative levels of Alexa Fluor 647-anti EGFR surface immunofluorescence (fold change of control) between control and DIE-NPs (*p* < 0.05). **** denotes the relative significance of Alexa Fluor 647-anti EGFR surface immunofluorescence (fold change of control) between DI-NPs and DIE-NPs (*p* < 0.05). Data are given as mean ± standard deviation (*n* = 3).

The in vitro targeting efficiency was verified (Figure 8A); bright-field (gray), Alexa Fluor 647 (red channel) and FITC (green channel) images of MG-63 cells treated with control, PLGA, Na^131^I, D-NPs, I-NPs, DI-NPs and DIE-NPs were collected by flow cytometer and surface immunofluorescence was calculated. Both the red and green channels indicate that the osteosarcoma cells incubated by the DOX loaded NPs (D-NPs) emitted a weak fluorescent signal and the I-131 NPs (DI-NPs) exhibited a slightly stronger fluorescence, and the targeted DIE-NPs emitted the strongest fluorescent signal. The red and green fluorescence images confirm specific cellular binding/uptake to the MG-63 cells. Furthermore, the Na^131^I, D-NPs, I-NPs, and DI-NPs fluorescence concentrations were limited to the cell fringe. In contrast, the targeted DIE-NPs uptake to the MG-63 cells is uniformly distributed throughout the MG-63 entire cytosol, suggesting the DIE-NPs penetrate the MG-63 cells mainly by receptor-mediated endocytosis and receptor-mediated membrane fusion pathways. Additionally, the flow cytometer (Figure 8B) displays the anti-EGFR labeled DIE-NPs treatment creating an extensive change in the fluorescence of the targeted MG-63 cells, confirming the DIE-NPs specific binding between the anti-EGFR targeting ligands and the targeted MG-63 cells. In contrast, the control (untreated) and PLGA revealed negligible fluorescence shifting. The mean fluorescent intensity is shown in Figure 8C, with Na^131^I ~480 a.u., D-NPs ~909 a.u., I-NPs ~1333 a.u., DI-NPs ~1527 a.u. and the targeted DIE-NPs ~57,908 a.u. The DIE-NPs emitted the strongest fluorescence intensity, and the relative EGFR surface immunofluorescence fold change of control is illustrated in Figure 8D, verifying that the anti-EGFR targeted DIE-NPs successfully achieved cellular binding/uptake to MG-63 cells.

### 3.11. Cytotoxicity Assay of 3D Spheroid MG-63 Osteosarcoma Cells, Live/Dead Cell Assay Method Using LionHeart FX Automated Live Cell Imager

**Figure 9:**
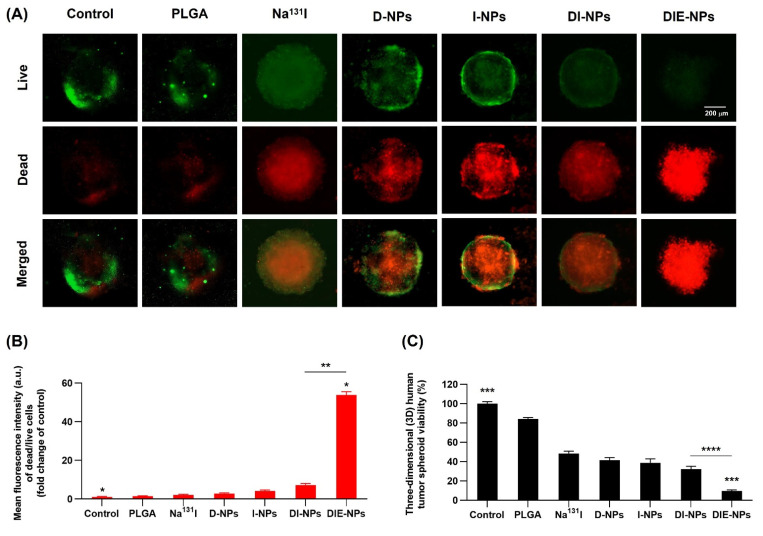
In vitro live/dead cell imaging of three-dimensional (3D) human tumor spheroids. (**A**) In vitro live/dead imaging of MG-63 cell tumor spheroid treated with control, PLGA, Na^131^I, D-NPs, I-NPs, DI-NPs and DIE-NPs. MG-63 cells were treated with a fixed concentration of I-131 activity 3.70 MBq for 72 h. Two-color fluorescence live (green channel) and dead (red channel) enabled the evaluation of live and dead cells to determine cell viability and cytotoxicity. Additionally, to eliminate out-of-focus fluorescent signals, all the channels were deconvolved by software [10× images, scale bar = 200 µm]. (**B**) Mean fluorescence intensity of dead/live (fold change of control) of MG-63 cell tumor spheroid treated with control, PLGA, Na^131^I, D-NPs, I-NPs, DI-NPs and DIE-NPs. MG-63 cells were treated with a fixed concentration of I-131 activity 3.70 MBq for 72 h. * denotes significance in mean fluorescence intensity of dead/live (fold change of control) of MG-63 cell tumor spheroid treatment between control and DIE-NPs (*p* < 0.05). ** denotes significance in mean fluorescence intensity of dead/live (fold change of control) of MG-63 cell tumor spheroid treatment between DI-NPs and DIE-NPs (*p* < 0.05). (**C**) Three-dimensional (3D) human tumor spheroid viability using Cell Titer-Glo^®^ 3D cell assay of MG-63 cell tumor spheroid treated with control, PLGA, Na^131^I, D-NPs, I-NPs, DI-NPs and DIE-NPs. MG-63 cells were treated with a fixed concentration of I-131 activity 3.70 MBq for 72 h. *** denotes significance in viability of MG-63 cell tumor spheroid treatment between control and DIE-NPs (*p* < 0.05). **** denotes significance in viability of MG-63 cell tumor spheroid treatment between DI-NPs and DIE-NPs (*p* < 0.05). Data are given as mean ± standard deviation (*n* = 3).

Figure 9A illustrates the fluorescence intensity (a.u.) of the dead cells (red channel) and live cells (green channel) in MG-63 spheroids and spheroid viability. At 72 h incubation, the 5000 cells treated by the non-targeted DI-NPs’ mean fluorescence intensity of dead/live was ~7.2-fold change over the control, compared to the targeted DIE-NPs’ mean fluorescent intensity of ~54-fold change of control (Figure 9B). 

As a result, the cytotoxicity assay verified that the 5000 initial cells treated by the targeted DIE-NPs experienced a ~4-fold greater cytotoxicity than non-targeted NPs (DI-NPs). Additionally, the DIE-NPs co-administration of DOX and Na^131^I had a ~4.5-fold higher cytotoxicity than the DOX monotherapy (D-NPs) (Figure 9C).

### 3.12. Three-Dimensional (3D) Human MG-63 Tumor Spheroid DIE-NPs Penetration

**Figure 10:**
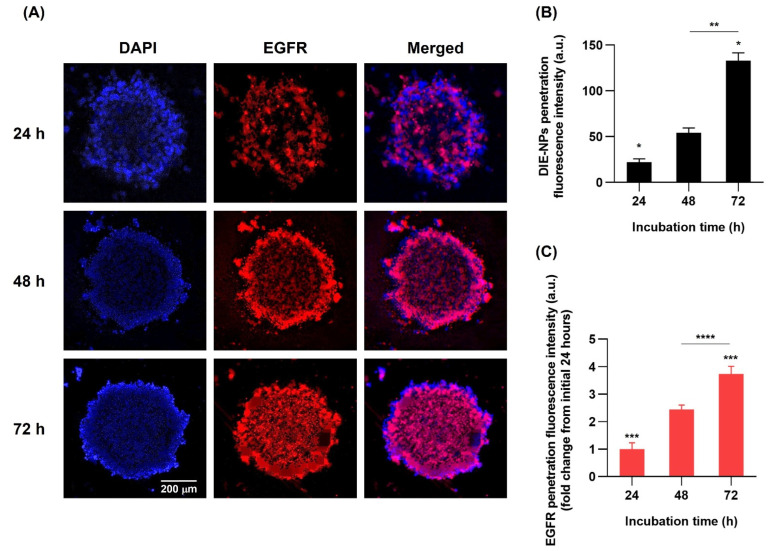
DIE-NPs penetration in a three-dimensional (3D) human MG-63 tumor spheroid. (**A**) Human MG-63 (EGFR+) spheroids fluorescence images treated with Alexa Fluor 647 anti-human EGFR antibody conjugated to the surface of the DIE-NPs (red channel), and MG-63 cell nucleus DAPI stained (blue channel) from initial 24 h at incubation times 24, 48 and 72 h. Scale bar = 200 µm. (**B**) Quantification of DIE-NPs penetration fluorescence intensity of Alexa Fluor 647 anti-human EGFR antibody conjugated to the surface of the DIE-NPs (red channel) from initial 24 h at incubation times 24, 48 and 72 h. * denotes significance in DIE-NPs penetration fluorescence intensity of Alexa Fluor 647 anti-human EGFR antibody conjugated to the surface of the DIE-NPs between initial 24 h and 72 h incubation times (*p* < 0.05). ** denotes significance in DIE-NPs penetration fluorescence intensity of Alexa Fluor 647 anti-human EGFR antibody conjugated to the surface of the DIE-NPs between initial 48 h and 72 h incubation times (*p* < 0.05). (**C**) The penetration fluorescent intensity (fold change from initial 24 h) of Alexa Fluor 647 anti-human EGFR antibody conjugated to the surface of the DIE-NPs (red channel) at incubation times 24, 48 and 72 h. *** denotes significance in penetration fluorescent intensity (fold change from initial 24 h) between initial 24 h and 72 h incubation times (*p* < 0.05). **** denotes significance in penetration fluorescent intensity (fold change from initial 24 h) between initial 48 h and 72 h incubation times (*p* < 0.05). Data are given as mean ± standard deviation (*n* = 3).

DIE-NPs penetration in a three-dimensional (3D) human MG-63 tumor spheroid to imitate an osteosarcoma dense tumor microenvironment (TME) enables an understanding of doxorubicin penetration [29]. Figure 10A illustrates the DIE-NPs penetration at 24, 48 and 72 h., validating the DIE-NPs penetration, and its role as a significant factor in determining a therapeutic effect. As a result, this study suggests the DIE-NPs achieved good penetration of the human MG-63 tumor spheroid with a fluorescence intensity of 22 a.u. at 24 h, increasing to 54 a.u. at 48 h and 133 a.u. at 72 h, suggesting penetration increased with time (Figure 10B). In addition, the fold increase in fluorescence intensity, which is the fold change from the initial 24 h reveals an increase from 1-fold at 24 h to 3.74-fold at 72 h (Figure 10C).

### 3.13. Cell Cycle

**Figure 11:**
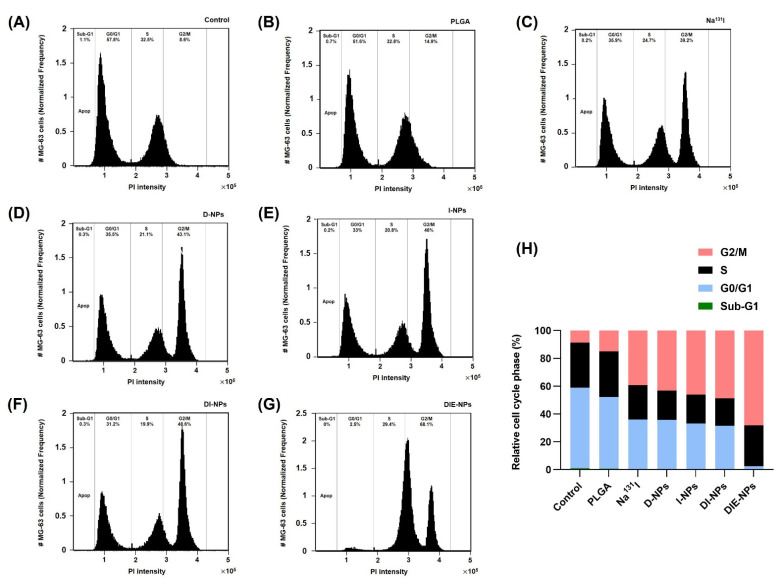
Cell cycle analysis. The number of MG-63 cells (normalized frequency) was assessed using ImageStreamX Mk II flow cytometer after the cell pellets were suspended in the Propidium Iodide (PI) staining solution. The time-dependent impact of the combination formulations on cell cycle progression determined the number of MG-63 in sub-G1, G0/G1, S, and G2/M treated. (**A**) Control. (**B**) PLGA. (**C**) Na^131^I. (**D**) D-NPs. (**E**) I-NPs. (**F**) DI-NPs. (**G**) DIE-NPs. (**H**) Relative cell cycle phase percentages of MG-63 in sub-G1, G0/G1, S, and G2/M treated control, PLGA, Na^131^I, D-NPs, I-NPs, DI-NPs, and DIE-NPs.

By examining the DNA content of the treated cells using flow cytometer, the time-dependent impact of the combination formulations on cell cycle progression was determined (Figure 11A–G). Figure 11H shows the MG-63 cell cycle distribution percentages after each treatment. The control (untreated) MG-63 cells control cell cycle profile of ~58% of the cells distributed in the G0/G1 phase, 33% in the S phase, and ~9% of the cells in the G2/M phase is shown in Figure 11A, whereas Figure 11G shows that the targeted multi-therapeutic DIE-NPs cell cycle has a G0/G1 phase of ~3%, S phase of ~29%, and G2/M phase of ~68%. This suggests that the DIE-NPs treatment resulted in a more significant cell population in the G2/M phase, and a reduction in cells in the G0/G1 phase in comparison to the control.

### 3.14. Blood Compatibility of Samples: Hemolysis Assay

**Figure 12:**
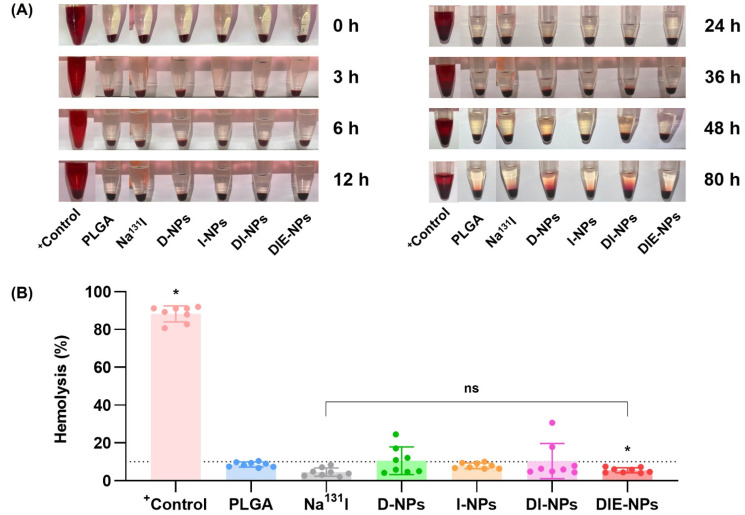
Nanoparticle in vitro hemolytic properties analysis: 8 × 10^9^ red blood cells/mL incubated at 37 °C. (**A**) Analysis of red blood cell pellets and the supernatant analysis for lysed hemoglobin; DMSO (positive control), PLGA, Na^131^I, D-NPs, I-NPs, DI-NPs and DIE-NPs after 0, 3, 6, 12, 24, 36, 48, and 80 h incubation. (**B**) Quantitative hemolysis percentage of the supernatant analysis for lysed hemoglobin; DMSO (positive control), PLGA, Na^131^I, D-NPs, I-NPs, DI-NPs after 0, 3, 6, 12, 24, 36, 48, and 80 h incubation. ns = no statistically significant difference in the hemolytic properties between Na^131^I and DIE-NPs (*p* > 0.05). * denotes significance in the hemolytic properties between DMSO (positive control) and DIE-NPs (*p* < 0.05). Data are given as mean ± standard deviation (*n* = 3).

A hemolysis assay was performed to evaluate the in vitro hemolytic potential damage from the functionalized DIE-NPs (Figure 12A). Using DMSO as a positive control, it was determined that the hemolysis was ~83% at 0 h and ~88% at 80 h (Figure 12B). In addition, the Na^131^I hemolysis was ~2% at 0 h, rising to ~8% at 80 h, indicating it initiated negligible damage to red blood cell membranes. Additionally, the I-NPs hemolysis was ~4% at 0 h, rising to ~10% at 80 h. The hemolysis of the D-NPs at 0 h was ~4%, and ~24% at 80 h, increasing to ~20%. Hemolysis of the DI-NPs was ~4% at 0 h, rising to 31% at 80 h. Furthermore, the DIE-NPs hemolysis at 0 h was ~7% and ~8% at 80 h. Additionally, 10% is considered a low percentage hemolysis [30,31,32]. As a result, the hemolysis assay determined that the anti-EGFR functionalized DIE-NPs are biocompatible with the red blood cell membranes blood with no adverse effects. 

### 3.15. Cellular Uptake by Macrophage Cells

**Figure 13:**
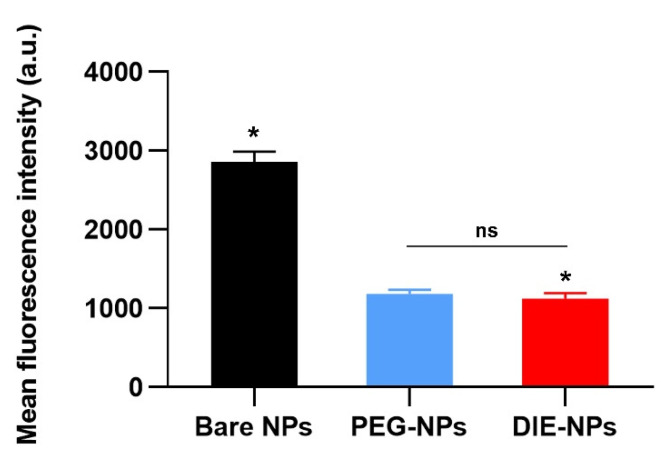
Cellular uptake by macrophage cells of non-coated bare PLGA nanoparticles (Bare NPs) and the stealth functionality of DIE-NPs in comparison to PEGylated nanoparticles (PEG-NPs). ns = no statistically significant difference in mean fluorescence intensity between PEG-NPs and DIE-NPs (*p* > 0.05). * denotes significance in mean fluorescence intensity between Bare NPs and DIE-NPs (*p* < 0.05). Data are given as mean ± standard deviation (*n* = 3).

The cellular uptake by macrophage cells (RAW 264.7 cells) was demonstrated for non-coated bare PLGA nanoparticles (Bare NPs) and the stealth functionality of DIE-NPs in comparison to PEGylated nanoparticles (PEG-NPs), the current gold standard for long-circulating nanoparticles (Figure 13). We discovered that the macrophage uptake of DIE-NPs is significantly lower than Bare NPs. Moreover, macrophage uptake of the DIE-NPs is comparable to that of the PEG-NPs, which is the current gold standard. The results confirm that the DIE-NPs are able to evade the immune system [33,34].

## 4. Discussion

The human MG-63 targeted nanotherapeutic drug delivery platform incorporating an anti-EGFR functionalized Na^131^I radiolabeled PLGA nanotherapeutic (DIE-NPs) is illustrated in Figure 1. In brief, the construction of the nanoplatforms involves 2 phases: (1) PEG-PLGA NPs synthesis by double emulsion and PLGA NPs radiolabeling with Na^131^I and doxorubicin loading into the PLGA core; (2) Conjugation of the anti-EGFR targeting ligand to the PLGA NPs.

PLGA, a copolymer of poly(lactic acid) and poly(glycolic acid), is a well-defined biomaterial with FDA approval for drug encapsulation because for drug release, it has excellent biodegradability, biocompatibility, and controllability [35]. Therefore, to fabricate the DOX loaded PLGA NPs (D-NPs), DOX was added to the NPs core by the double emulsion method during the inner phase. Similarly, for the radiolabeled NPs (I-NPs), the hydrophilic Na^131^I was added at the inner phase, to remove the necessity of using chelating agents. As a consequence, trapping the Na^131^I radiometal into the PLGA core enables greater radioactivity doses to be delivered than nanocarriers that are externally surface labeled, and thus deliver higher cytotoxicity [36]. Likewise, for the DI-NPs, the combined DOX and Na^131^I were added at the inner phase, and the non-chelator method was performed to integrate Na^131^I into the PLGA core at the inner phase. Moreover, excluding bulky chelator molecules conserves the PLGA NPs integrity [37]. Furthermore, the DIE-NPs incorporate DOX and Na^131^I into the core, with the addition of anti-human EGFR Alexa Fluor 647 antibody conjugated to the DIE-NPs surface, ~1125 molecules/NP (Table 1). It specifically binds to epidermal growth factor (EGF), transforming growth factor alpha, amphiregulin a transmembrane tyrosine kinase, betacellulin, and heparin-binding EGF-like growth factor for targeted receptor-mediated endocytosis. Increasingly PEGylated phospholipids are applied for drug delivery systems, and the Food and Drug Administration has approved DSPE−PEG(2000)−COOH for use in medical applications [38]. Moreover, chemical coupling techniques readily enable the attachment of targeting moieties to phospholipid−PEG conjugates. PEGylated phospholipids and targeting moieties can be conjugated in one step. Additionally, phospholipid−PEG−ligand conjugates can serve as a self-assembling nanocarrier. In addition, the insertion of targeting moieties on PEGylated phospholipid-decorated NPs does not significantly alter their physicochemical properties. As a result, the attachment of targeting moieties can enhance targeting by several fold compared to non-targeted NPs [39]. As illustrated in Figure 1, the hydrophilic and lipophilic properties of DSPE−PEG(2000)−COOH form a stealth layer around the outer shell of the PLGA NPs, prolonging NPs circulation time in the body and facilitating sustained drug release (Figure 4C and Figure 6B) [40]. Furthermore, it has been reported that the activated PEG terminal groups boost tissue selectivity and transport efficiency when connected to the anti-EGFR targeting ligands on the PLGA NPs surface [41,42]. Additionally, studies have indicated that PEG significantly inhibits non-specific protein adsorption, benefiting passive and active targeting [43,44,45].

Furthermore, characterization of the nanoparticles determined the targeted radiolabeled DIE-NPs diameter as ~175 nm (Figure 3A); this is within the size range of nanocarriers with a diameter of less than 200 nm that can extravasate from blood arteries into tumor tissue as a consequence of the leaky vasculature and decreased lymphatic drainage. In addition, a number of investigations have shown that NPs with a diameter of 100–200 nm are the most effective for theranostic use [46]. Figure 3B illustrates the DIE-NPs polydispersity (PDI) of 0.15, which is homogenous and considered acceptable for biomedical use [47]. Additionally, the DIE-NPs have a zeta potential of −59 mV (Figure 3C), as both the poly(lactic-co-glycolic acid) and EGFR antibodies have a negative charge. The zeta potential stability over 96 h confirms that the DIE-NPs have good stability. NPs having a zeta potential higher than or equal to +30 mV or less than −30 mV are deemed to have a sufficient repulsive force for improved particle size and colloidal physical stability [48,49]. In addition, it was reported by Elvin Blanco et al. that the zeta potential of a nanoparticle is a property that enables it to pass through the cell membrane, and negatively charged NPs impede elimination by the kidney and reticuloendothelial system (RES) [50]. Furthermore, Figure 3E shows the chemical purity of the DIE-NPs, confirming that the Na^131^I radiochemical purity is greater than 95%, and complies with the World Health Organization Consultation Document standard [25]. In addition, the Na^131^I radiolabeling yield (Figure 3F) verifies that over a 24 h period, the radiolabeling yield was over 95%. 

Notably, epidermal growth factor receptor (EGFR) is reported to be overexpressed in osteosarcoma and, for that reason, is targeted by a human EGFR antibody conjugated to our novel targeted multifunctional radio-nanotherapeutic [51]. Moreover, the overexpression of the EGF tyrosine kinase stimulates tumor survival, growth, angiogenesis, invasion and metastatic spread [52]. Furthermore, it stimulates the MAPK/ERK and PI3K/Akt pathways in osteosarcoma, resulting in cytoskeleton restructuring and increased cell proliferation and migration. Therefore, we used a human EGFR Alexa Fluor 647-conjugated antibody to recognize and specifically target proteins expressed on the surface of osteosarcoma tumor cells. In addition, the human EGFR antibody facilitates receptor-mediated endocytosis, resulting in enhanced therapeutic efficacy [53]. Recently, Wang et al. identified cell-surface antigens for targeted osteosarcoma therapy and established CD276, MT1-MMP, and MRC2 were overexpressed in osteosarcoma but not overexpressed in normal tissue [54]. Furthermore, another study found that EGFR expression in human osteosarcoma cell lines determined a greater expression of EGFR in MG-63, 143B, and MNNG-HOS cells and also an increase in EGFR mRNA level [55]. It has also been shown that inhibiting EGFR in human osteosarcoma cell lines may effectively reduce cell migration, invasion, and colony formation in these cells [56]. From the results (Figure 7), it can be observed that the MG-63 targeted DIE-NPs fluorescence intensity indicated ~80-fold greater targeting efficacy to MG-63 cells (EGFR+) than fibroblast cells (EGFR−), confirming that the EGFR targeted DIE-NPs specifically targets MG-63 cells. In addition, the cellular binding/uptake results indicate that the EGFR-overexpressed MG-63 cells were significantly internalized, exhibiting a strong green and red fluorescence, with a significantly higher mean fluorescence intensity (green and red channel) of the EGFR targeted DIE-NPs than the DI-NPs, corroborating EGFR overexpression as a potential target (Figure 8).

Furthermore, standard cytotoxic chemotherapy for cancer is frequently immunosuppressive and linked to drug resistance and tumor recurrence. Nevertheless, certain cytotoxic cancer chemotherapeutic drugs, such as doxorubicin, can kill tumor cells via an immunogenic cell death pathway, resulting in the activation of a strong adaptive and innate immune anti-tumor response, which has the potential to enhance the efficacy of chemotherapy significantly. Additionally, Casares et al. reported that anthracyclines are capable of eliciting immunogenic cell death in vitro, ex vivo, and in vivo [57]. In addition, doxorubicin has the ability to upregulate cancer cell programmed death-ligand 1 (PD-L1) expression through the initiation of danger signals, stimulating antitumor immunogenicity, through the triggering of cytotoxic T lymphocytes, antigen-presenting cells development, increase in myeloid-derived suppressor cells and or immunosuppressive regulatory T cell reduction [58]. Furthermore, research by Wang et al. reported that the incorporation of DOX in nanoparticles could control drug release, improve the encapsulation of the drug, and reduce drug side effects [59]. In another study, Kim et al. developed PLGA NPs loaded with DOX (DP-NPs) to induce immunogenic cell death (ICD) and provided controlled DOX release over 14 days [60]. Their platform effectively triggered ICD and decreased cell viability in vitro, and the DOX-NP significantly inhibited tumor development with fewer side effects. Moreover, the tumor included far more mature dendritic cells and cytotoxic T lymphocytes, suggesting an improved antitumor immune response. 

However, the use of DOX has limits because it lacks tumor selectivity, has dose-dependent cardiotoxicity, and increases drug resistance. Additionally, because of cancer heterogeneity, the administration of a single chemotherapeutic can be inadequate to destroy malignant cancer cells [61]. Nonetheless, to overcome these obstacles, we designed a nanocarrier with good specificity to MG-63 cells, enabling the release of a good DOX concentration and Na^131^I to the MG-63 spheroids over 7 days. Furthermore, the DOX release kinetics can be readily regulated by altering the polymer characteristics [62]. Increasing the lactide and glycolide ratio can improve the hydrophobicity of PLGA, resulting in a slower rate of degradation and slower drug release [63]. The nanoparticle formulations in this study used a carboxy-terminated PLGA polymer 0.66 dL/g PLGA with a 4.7 kDa molecular weight and a 50:50 ratio of lactic acid and glycolic acid; these have a good rate of degradation and are regularly used for PLGA drug delivery nanocarriers. Kumskova et al. studied the influence of PLGA molecular weights on doxorubicin release and found that 4.7 kDa resulted in significantly higher rates of DOX release and quicker polymer degradation [62]. Additionally, with a 50:50 ratio with higher hydrophilic properties of glycolic acid, the preferential degradation results in quicker PLGA degradation as the lactide PLGA copolymers with lower hydrophilicity absorb less water and decay more slowly [64]. The DOX initial weight was varied in this study, and the DOX encapsulation efficiency and drug release were studied. It was determined that 5 wt% of DOX achieved an optimum encapsulation efficiency of ~85% and this was applied for subsequent experiments. In addition, 5 wt% DOX loading (5 × 10^−4^ µg/mL), which achieved an encapsulation efficacy of ~85% and achieved IC_50_, was the optimal formulation (Figure 5). 

Furthermore, due to the increase in DOX solubility at moderately acidic pH levels, the pH environment influences the stability of DOX, with a quicker release occurring at slightly acidic pH, possibly owing to the greater solubility of DOX in an acidic environment [65]. Moreover, the release of DOX from the PLGA matrix results from bulk diffusion, desorption, surface diffusion, and matrix degradation, which significantly affects the DOX release. Therefore, drug release profiles from PLGA NPs can be adjusted and is dependent on the particle size and the drug quantity loaded [35]. A usual drug release profile from PLGA nanoparticles comprises a burst phase initially, followed by an induction period, then a slower drug release phase and a final drug release phase. In this study, the DIE-NPs drug initial release phase resulted in ~20% of the DOX release; ~49% at 24 h, ~62% at 48 h and 84% at 120 h (Figure 6B), indicating the DOX has sustained drug release kinetics, with no burst-like drug kinetics over 120 h. The cumulative drug release study over 120 h ascertained that approximately ~53% of DOX was released within the first 36 h, and approximately 80% of DOX was released over the course of 120 h, indicating the majority of the drug release, with 72% occurring within the first 72 h. The prolonged DOX release is due to the slow diffusion of drug molecules through the matrix of PLGA nanoparticles as well as the DSPE−PEG(2000)−COOH coating, forming an additional diffusional layer. Suk et al. have previously reported this phenomenon [44].

Notably, the co-administration of anticancer agents or therapeutics is often considered a viable strategy for cancer treatment to reduce the dose of each drug [66], reducing the drug’s dose-dependent toxicity in non-target normal cells/tissues, leading to an improved clinical outcome and fewer side effects. Anthracyclines and some forms of radiation therapy can initiate immunogenic cell death. The evidence suggests that the conventional anticancer drug DOX, an anthracycline antibiotic, not only triggers tumor cell death by apoptosis but also evokes effective antitumor immunity responses by inducing immunogenic cell death [67]. Furthermore, combining the delivery of DOX and Na^131^I in this study enables the targeting of several cellular pathways simultaneously to inhibit drug-resistant cell population survival [68]. Na^131^I is extremely radioactive and has a short half-life of 8 days, decaying mostly via beta-emission (606 keV; 90%), with a soft tissue penetration of ~1 mm. In addition, there is a low linear energy transfer of approximately 0.25 keV/m because the beta energy absorption is close to the radiation source, making Na^131^I an effective therapy for cancer tumors. Additionally, Na^131^I generates high-energy gamma radiation (364 keV; 10%), which is used for diagnostic imaging. Therefore, the Na^131^I provides the DIE-NPs with the theranostic capabilities of therapy and diagnosis [69].

The results from the 3D cytotoxicity assay (Figure 9), ascertained the DIE-NPs co-administration of DOX and Na^131^I by the targeted DIE-NPs has a ~4-fold greater cytotoxicity than non-targeted NPs (DI-NPs). Additionally, the DIE-NPs co-administration of DOX and Na^131^I had a ~4.5-fold higher cytotoxicity than the DOX monotherapy (D-NPs) (Figure 9C). Therefore, the 3D cytotoxicity confirms that the co-administration of DIE-NPs has significantly greater cytotoxicity on MG-63 cells. Similarly, it was reported that combination therapy for gliomas using doxorubicin-loaded, ^131^I-labeled nanoliposomes resulted in significantly longer survival of mice and lesser tumor size than monotherapy [70]. Likewise, Li et al. targeted EGFR overexpression in various cancer cells with an anti-epidermal growth factor receptor-targeted ^131^I-labeled nanoparticle with a specific radioactivity of 370–690 MBq/mg for their EGFR targeted NPs and non-targeted NPs, resulting in a ~50–85% labeling efficiency [71] in comparison to the labeling efficiency in this study of 98% (Figure 3 and Figure 7).

The tumor microenvironment (TME) is diverse in nature, consisting mostly of an extracellular matrix (ECM) and a stroma comprised of fibroblasts, adipocytes, endothelial cells, immune cells, and a variety of cytokines and growth hormones. Additionally, the ECM’s microarchitecture, composition, stiffness, and topography all play a critical role in tumor growth and directly impact cell behavior. Multicellular spheroids in 3D cell cultures have biostructural and biofunctional features that are more similar to those of biological tissues in vivo than in 2D planar cell cultures [72]. Furthermore, multicellular spheroids often exhibit distinct properties from monolayer cells, including ECM deposition, growth factor secretion, gene expression patterns [73] and imitate the TME, providing insight into tumor growth, progression, and related molecular pathways in cancer therapy [74]. Multicellular spheroids replicate the bulk of characteristics of in vivo human solid tumors and, thus, their treatment resistance [75]. A 3D bioprinted osteosarcoma model by Lin et al. demonstrated the model had sensitivity to autophagy pathway targeted therapy and provided different drug sensitivities and cell metabolic features as a result of the simulated extracellular matrix [76]. Additionally, 3D multicellular spheroids are usually cultivated using either the liquid overlay technique or the spinner cell culture method. In this study, the liquid overlay technique was utilized with Corning ultra-low attachment plates, which inhibit MG-63 cells from adhering to the well surface, promoting cell aggregation and the production of homogenous floating spheroids. The MG-63 spheroids in this study were ~500 µm in diameter and can imitate various features seen in solid human tumors ranging in size from 0.5 to 1 mm^3^ (Figure 6, Figure 9 and Figure 10). These features have an impact on the therapeutic efficacy of different medicines and other pharmacological compounds on spheroids through processes similar to those identified in solid human tumors in vivo [77].

Furthermore, the cell cycle progression established that the DIE-NPs treatment significantly reduced the cell population in the G0/G1 quiescence and growth phase where organelles duplicate. G1 is the first checkpoint and ensures there are no cell or DNA irregularities. The cells recognize when they are not developing normally, and depending on the severity of the problem, the cell may repair itself or initiate apoptosis. The co-administration of DOX and Na^131^I disrupts DNA repair and generates free radicals resulting in DNA damage, and triggers the activation of apoptosis pathways, inhibiting MG-63 cell proliferation and promoting cell cycle arrest in the G0/G1 phase [78,79]. One of the processes by which I-131 radiation inhibits cell proliferation is apoptosis, and the other is radiation-induced cell reproductive failure. Both of these processes lead to cell death (programmed cell death) (Figure 11) [80]. 

According to several studies conducted in vitro, a percentage of hemolysis ranging from 5 to 25% is considered “of no concern” [30,31,32,81,82]. The PEGylated anti-EGFR functionalized DIE-NPs were found to be biocompatible with red blood cells and to have no adverse effects (Figure 12). Furthermore, the Figure 13 illustrates that the DIE-NPs were found to have considerably lower macrophage uptake than the bare PLGA NPs. Additionally, the macrophage uptake of the DIE-NPs is equivalent to that of the PEGylated NPs, the current gold standard [83].

## 5. Conclusions

The combination therapy of Na^131^I-labeled and DOX-loaded PLGA nanoparticles is the first study to research the effects of treating MG-63 osteosarcoma with the co-administration of DOX and Na^131^I in an anti-EGFR targeted PLGA nanotherapeutic. The PLGA core has excellent biocompatibility and degradation properties and is deemed safe for the biomedical applications. In addition, the nanoparticles containing 5 wt% of initial drug input had a ~85% drug encapsulation efficiency and a 3% drug loading efficiency. This enabled persistent DOX release, with ~53% of the DOX release occurring within the first 36 h, and ~80 of DOX total release, over 5 days. The EGFR-targeted radiolabeled nanoparticle DIE-NPs exhibited greater MG-63 cellular binding and uptake in comparison to the non-targeted D-NPs, achieving ~64-fold higher binding/uptake to MG-63 cells than the non-targeted D-NPs, and ~80-fold higher targeting efficacy to MG-63 cells (EGFR+) than fibroblast cells (EGFR−), with a statistically significant difference. Furthermore, the DIE-NPs EGFR-targeted radioactive nanotherapeutic exhibited good penetration and uptake of Na^131^I to the 3D MG-63 spheroids. Those treated by the targeted DIE-NPs have a ~4-fold greater cytotoxicity than non-targeted NPs (DI-NPs). Additionally, the DIE-NPs co-administration of DOX and Na^131^I had a ~4.5-fold higher cytotoxicity than the DOX monotherapy (D-NPs), with a statistically significant difference. The co-administration of DOX and Na^131^I (DIE-NPs) disrupts DNA repair and generates free radicals resulting in DNA damage, and triggers the activation of apoptosis pathways, inhibiting MG-63 cell proliferation and promoting cell cycle arrest in the G0/G1 phase. Furthermore, the PEGylated anti-EGFR functionalized DIE-NPs resulted in minimal uptake by macrophage cells, and were found to be biocompatible with red blood cells and to have no adverse effects.

Our long-term research aims are to enhance this nanotherapeutic for clinical use. However, various elements, such as large-scale synthesis, clinical safety profile, and the long-term consequences of bone-seeking nanomedicines need addressing prior to converting this platform to human application. This theranostic nanotherapeutic has great potential to be further enhanced for individualized osteosarcoma therapy in the future.

## Figures and Tables

**Table 1:** Quantification of nanoparticle-bound ligands. The surface density of nanoparticles conjugated with DSPE−PEG(2000)−COOH was measured at various ligand weights and anti-human EGFR antibody 1 µg. The surface coverage, molecules/mg NP, and the molecules per NP were determined using surface area and density approximations for the spherical PLGA nanoparticles. Data represent mean ± standard deviation (*n* = 3).

Ligand	Ligand (µg)	Surface Density (nmol/mg NP)	Surface Coverage (nmol/cm^2^ NP)	Molecules/mg NP	Molecules/NP
DSPE−PEG(2000) −COOH	25	9 ± 2	7 ± 3	5.41 × 10^15^	36 ± 5
	50	19 ± 5	15 ± 4	1.08 × 10^16^	75 ± 8
	100	34 ± 6	33 ± 7	2.17 × 10^16^	148 ± 18
	250	92 ± 11	77 ± 8	5.41 × 10^16^	363 ± 17
	500	180 ± 23	150 ± 17	1.08 × 10^17^	719 ± 11
	1000	360 ± 38	300 ± 29	2.17 × 10^17^	1439 ± 59
anti-human EGFR antibody	1	7.46 × 10^−3^	6.22 × 10^−3^	4.5 × 10^12^	1125 ± 23

## Data Availability

The data presented in this work are contained within the article.
